# Successful Removal of Large Intraocular Foreign Body by 25-Gauge Microincision Vitrectomy Surgery

**DOI:** 10.1155/2011/940323

**Published:** 2011-04-04

**Authors:** Hiroshi Kunikata, Megumi Uematsu, Toru Nakazawa, Nobuo Fuse

**Affiliations:** ^1^Department of Ophthalmology and Visual Science, Tohoku University Graduate School of Medicine, 1-1 Seiryo-machi, Aoba-ku, Sendai 980-8574, Japan; ^2^Division of Visual Advanced Medicine, Tohoku University Graduate School of Medicine, Sendai 980-8574, Japan

## Abstract

We describe a new technique for removing a large intraocular foreign body by 25-gauge microincision vitrectomy surgery (25G-MIVS). Noncomparative interventional case series were performed at a single centre. Two patients with a long smooth intraocular vitreal foreign body underwent phacoemulsification and aspiration, intraocular lens implantation, 25G-MIVS, and extraction of the foreign body. The foreign body was removed through a posterior capsulorhexis, anterior continuous curvilinear capsulorhexis, and a corneal incision. In both cases, the foreign body was safely removed through the corneal incision, and IOL was implanted and well positioned. The surgical incision did not require suturing. No postoperative complications associated with this technique were found. The corneal endothelial cell density was maintained over 2000 cells/mm^2^ in both cases during recent follow-up examinations. Our findings indicate that 25G-MIVS with this technique can be used to extract a long slender smooth foreign body. It is safe, without complications, and can be performed without enlarging the 25-gauge sclerotomy.

## 1. Introduction

The removal of an intraocular foreign body is difficult, and less invasive techniques that lead to good postoperative vision from the early stage are being investigated. If a large foreign body is extracted from the eye, an enlargement of the sclerotomy is needed, and intraoperative suturing is required. The suturing usually leads to corneal astigmatism. 

25-gauge microincision vitrectomy surgery (25G-MIVS) was first reported in 2002, and this technique is commonly used worldwide for various retinal diseases including rhegmatogenous retinal detachments [1–4]. The increase in the use of MIVS has been enhanced by studies that demonstrated significant reductions in postoperative astigmatism, conjunctival injection, pain, and discomfort [5–7]. However, the use of 25G-MIVS for the removal of a foreign body without an enlargement of the sclerotomy had not been reported [8, 9]. 

The purpose of this study was to determine whether 25G-MIVS can be used to remove an intraocular foreign body without suturing.

## 2. Technique

**Case 1**

**Figure 1:**
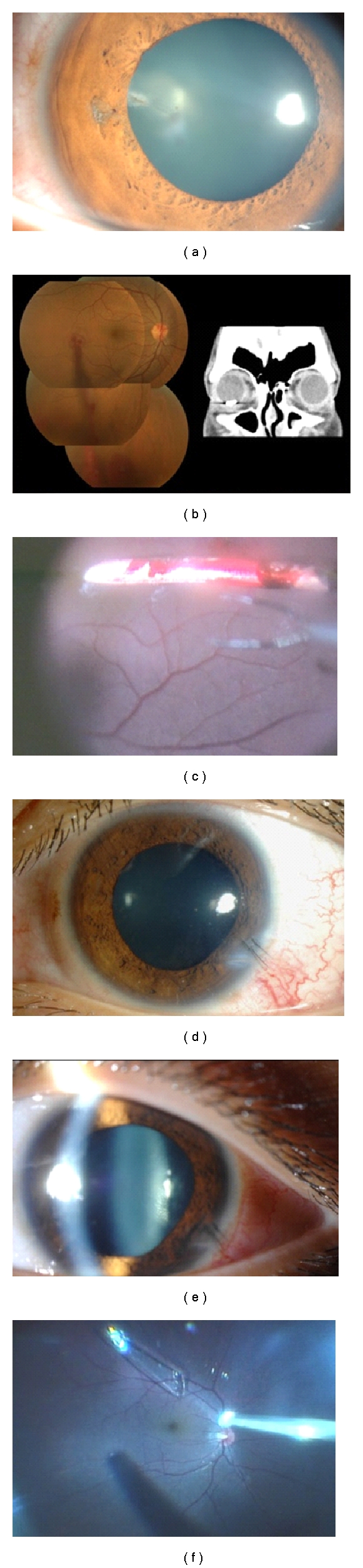
Preoperative slit-lamp photographs, preoperative fundus photograph, preoperative computed tomographic image, and intraoperative photographs of intraocular foreign body (Case 1; (a, b, c), Case 2; (d, e, f)). (a) Preoperative slit-lamp photograph shows a slight penetrating wound in the iris and lens at the 9 o'clock position and a posterior subcapsular cataract at the same position. (b) Fundus photograph showing vitreous haemorrhage and retinal tear with subretinal haemorrhage located on the temporal side of the macula. Computed tomographic image showing a large foreign body. (c) Intraoperative fundus showing a large metallic intraocular foreign body anterior to the retina. (d) External photograph showing the penetrating wound at the 4 o'clock position and the corneal wound was closed by corneal sutures during the initial surgery. (e) Slit-lamp photograph showing that the posterior subcapsular cataract has progressed. (f) Intraoperative fundus photograph showing large glass intraocular foreign body anterior to the retina.

A 31-year-old man presented 4 days after a corneal laceration in the temporal area of the right eye (Figure 1(a)). His best-corrected visual acuity (BCVA) was 6/20, and the intraocular pressure was normal. The corneal wound was self-sealed without any leakage, but a small penetrating wound was seen in his right iris at the 9 o'clock position. Slit-lamp examination showed a posterior subcapsular cataract at the same position. Fundus examination showed vitreous haemorrhage, and computed tomography showed a metallic foreign body in the vitreous (Figure 1(b)).

**Figure 2:**
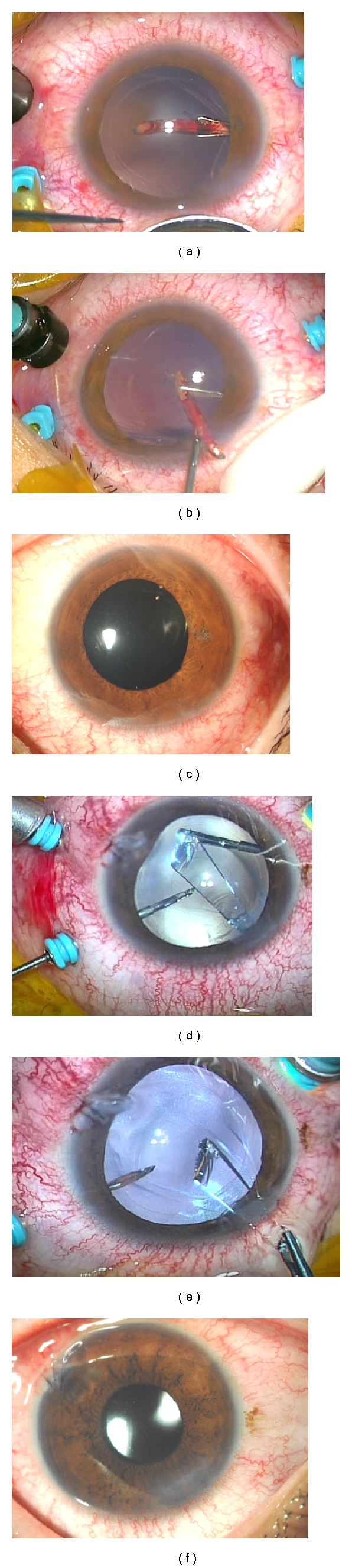
Intraoperative photographs and postoperative slit-lamp photographs of Case 1 (a, b, c) and Case 2 (d, e, f). (a) and (b) Metallic foreign body extracted through an anterior and posterior capsulorhexis, and corneal incision (triple C-through technique). (c) Slit-lamp photograph (inverted image as seen by the surgeon) 1 day postoperatively showing no need of suturing, no subconjunctival haemorrhage, and well-positioned intraocular lens. (d) and (e) Glass foreign body extracted through an anterior and posterior capsulorhexis, and corneal incision (triple C-through technique). (f) Slit-lamp photograph (inverted image as seen by the surgeon) 1 day postoperatively showing no need of suturing, except the original penetration wound, no subconjunctival haemorrhage and well-positioned intraocular lens.

The foreign body was a straight metallic nail without a head that was 1.0 mm in diameter and 7.0 mm long (Figure 1(c)). We performed phacoemulsification and aspiration (PEA) through a 2.4 mm corneal incision, 25G-MIVS, and extracted the foreign body. First, we picked up the foreign body off of the retina with forceps and moved it into the vitreous cavity. Then, it was moved into the anterior chamber through a posterior capsulorhexis and an anterior continuous curvilinear capsulorhexis, and we grasped the foreign body with another forceps and removed the foreign body through the corneal wound which was used for PEA. Thus, the foreign body was extracted through a posterior capsulorhexis, an anterior continuous curvilinear capsulorhexis, and the corneal incision (triple C-through technique; Figures 2(a) and 2(b)). In addition, endophotocoagulation was performed on a retinal tear and on the area surrounding a subretinal haemorrhage located on the temporal side of the macula. An intraocular lens (IOL) was implanted in the capsular bag. All wounds including the incision for the cataract and vitreous surgeries did not require any suturing, and the IOL was well positioned (Figure 2(c)).

One month after the surgery, the BCVA was 20/20, and this BCVA was maintained for 32 months. No postoperative complications except a small epiretinal membrane developed during the 32 months of followup. The corneal endothelial cell density at baseline and at 32 months was 2834 and 2288 cells/mm^2^, respectively.

**Case 2**

A 21-year-old man presented with a 7-day-old corneal laceration at the 4 o'clock position of the left eye (Figure 1(d)). The wound was closed by the initial surgery, and there was a trace of a penetrating wound in the corresponding iris at same position. Slit-lamp examination showed a posterior subcapsular cataract (Figure 1(e)). Indirect ophthalmoscopy showed that the vitreous was clear, but there was a large glass-like object in the vitreous free from the retina (Figure 1(f)). The foreign body was a piece of glass that was 2.0 mm wide and 8.0 mm long. The retina around the foreign body was not inflamed. The BCVA was 20/20 in his left eye, and the intraocular pressure was normal.

We performed PEA through a 2.4 mm corneal incision, 25G-MIVS, extraction of the foreign body, and implantation of an IOL in the sulcus. Before grasping the foreign body with 25-gauge forceps, perfluorocarbon liquid (PFCL) was used to float the foreign body above the retina and macula. The floating foreign body was located at the margin of the PFCL because of its buoyancy, gravity, and PFCL's surface tensity, and we grasped the foreign body with forceps and removed it as in Case 1. Thus, the foreign body was extracted through a posterior capsulorhexis, an anterior continuous curvilinear capsulorhexis, and the corneal incision (triple C-through technique; Figures 2(d) and 2(e)). The 2.4 mm corneal incision was slightly enlarged to 3 mm to extract the foreign body safely. All of the surgical wounds including that for the cataract surgery required no suturing (Figure 2(f)).

One month after the surgery, the BCVA was 20/20, and this BCVA was maintained for 6 months, and no complications developed during the six-month followup. The corneal endothelial cell density at baseline and at 6 months was 2884 and 3021 cells/mm^2^, respectively.

## 3. Discussion

The removal of a foreign body usually requires a relatively large sclerotomy, and closing the sclerotomy with sutures often leads to postoperative corneal astigmatism. In addition, extracting a foreign body through the sclerotomy can damage the ciliary body and peripheral retina because it is difficult to see the foreign body when it is being extracted. Thus, we believe that extracting a foreign body through a small corneal wound that does not require suturing is a safer way to obtain good vision postoperatively. 

The extraction of a foreign body through a 6 mm sclerocorneal tunnel using 20-gauge conventional vitrectomy instruments was recently reported [8]; however, the scleral incision required suturing. The use of 25G-MIVS to remove foreign body has also been reported, although an enlargement of the sclerotomy was required in all cases [9]. We combined posterior capsulorhexis with microincision cataract surgery (corneal incision 2.4 mm) and vitreous surgery (25G-MIVS) as a safe method of extracting a foreign body without complications and not requiring suturing. 

Our study has several weaknesses, including its retrospective nature, only two cases, and short follow-up periods. However, we had very good results, and we recommend that a soft shell be used to protect the corneal endothelial surface and care be taken to keep the foreign body from touching the corneal endothelial surface. PFCL also should be used to protect the posterior part of retina for an accidental falling of the foreign body during this procedure. This technique of triple C-through technique is probably best suited to a long slender smooth foreign body and should not be used for larger foreign bodies of odd shape. 

In conclusion, under favorable conditions of intraocular foreign bodies, we recommend 25G-MIVS to remove foreign bodies safely without suturing. Further investigations including evaluation of the postoperative visual quality and complications are needed to determine efficacy of this procedure.

##  Disclosure

This paper was presented partially at the Annual Meeting of the 31th Japanese Society of Ophthalmic Surgeons, Yokohama, February 2008.
